# Network pharmacology and in vivo evidence of the pharmacological mechanism of geniposide in the treatment of atherosclerosis

**DOI:** 10.1186/s12906-024-04356-x

**Published:** 2024-01-24

**Authors:** Guiping Ma, Qinqin Dong, Feng Li, Zheng Jin, Jianbin Pi, Wei Wu, Junlong Li

**Affiliations:** 1 Beijing University of Chinese Medicine Affiliated Shenzhen Hospital, Shenzhen, China; 2 The First Affiliated Hospital of Guangzhou University of Traditional Chinese Medicine, Guangzhou University of Traditional Chinese Medicine, Guangzhou, 510405 China; 3 ZhuJiang Hospital, Southern Medical University, Guangzhou, China; 4 Foshan Hospital Traditional Chinese Medicine, Guangzhou University of Traditional Chinese Medicine, Foshan, China

**Keywords:** Atherosclerosis, Geniposide, Network pharmacology, Molecular docking, Inflammation

## Abstract

**Background:**

Atherosclerosis (AS) is a fundamental pathological state in various cardiovascular diseases. Geniposide, which is the main active component of *Gardenia jasminides*, is effective against AS. However, the underlying molecular mechanisms remain unclear. Here, we sought to elucidate them.

**Methods:**

The targets of AS and geniposide were collected from online public databases. The potential mechanism of Geniposide in treating AS was predicted by constructing a protein–protein interaction (PPI) network and conducting Gene Ontology (GO) and Kyoto Encyclopaedia of Genes and Genomes (KEGG) pathway enrichment analyses. Hub proteins and core pathways were verified by molecular docking and in vivo experiments. Moreover, the effect of geniposide on AS was assessed by measuring the atherosclerotic plaque area in the thoracic aorta of mice. ApoE^−/−^ mice were used to establish AS models and randomly divided into different groups. Two different doses of geniposide were administered to the mice. Hematoxylin and eosin (HE) staining was performed to evaluate the effects of geniposide on AS. Oil Red O and Sirius Red staining were used to evaluate plaque stability. The protein expression of key markers involved in the signalling pathways was examined using western blotting and immunofluorescence.

**Results:**

A total of 239 active targets, 3418 AS-related disease targets, and 129 overlapping targets were identified. Hub genes were detected, and molecular docking revealed that geniposide strongly interacted with hub proteins (AKT1, VEGFA, CTNNB1, MMP9, and EGFR). Moreover, 109 signalling pathways, including the Rap1 signalling pathway, were identified using enrichment analysis. The results of in vivo experiments demonstrated that geniposide reduced body weight and blood lipid levels, alleviated the formation of atherosclerotic plaques, enhanced plaque stability, and inhibited inflammation, at least partially, by activating the Rap1/PI3K/Akt signalling pathway in ApoE^−/−^ mice.

**Conclusion:**

Geniposide can alleviate AS and enhance the stability of atherosclerotic plaques by regulating the Rap1/PI3K/Akt signalling pathway.

**Supplementary Information:**

The online version contains supplementary material available at 10.1186/s12906-024-04356-x.

## Introduction

Atherosclerosis (AS) is the basic pathological state of cardiovascular disease and is characterised by lipid accumulation and narrowing of the arterial lumen [1–3]. AS is an important cause of most cardiovascular diseases, cerebral infarction, stroke, and peripheral vascular diseases, which seriously threaten patient health [4]. Lipids are essential at any stage of AS; therefore, AS is also considered a chronic lipid-related disease [5]. Lipid lowering is the cornerstone of the prevention of AS and its complications [6]. However, cardiovascular events still occur frequently even in individuals who strictly manage lifestyle and drug treatment to reach the low-density lipoprotein cholesterol (LDL-C) level recommended by the guidelines [7]. In addition to radical reduction of LDL-C levels and control of blood pressure, the approach to residual cardiovascular risk focuses on resolving systemic inflammation, hypertriglyceridaemia, and coagulation pathways [8]. Chronic inflammation is a decisive event in the progression of AS because it can cause the infiltration of multiple immune cells and the secretion of proinflammatory factors [9, 10]. Multiple agents beyond canakinumab and colchicine are being considered or under active investigation in AS trials, including direct upstream NLRP3 antagonists and downstream IL-6 inhibitors [8]. However, most cardiologists are not yet ready to treat patients with arteriosclerotic cardiovascular disease using a combination of lipid-lowering agents and anti-inflammatory drugs because the long-term risk–benefit profile of anti-inflammatory drug therapy has not been properly defined [7]. Therefore, alternative therapies that inhibit inflammation should be actively investigated to treat AS.

Geniposide is an iridoid glycoside isolated from gardenia, which is used in traditional Chinese medicine (TCM), and the main active component of *Gardenia jasminides* [11]. Previous studies have reported that geniposide has potential curative effects in various diseases, such as diabetic nephropathy [12], non-alcoholic fatty liver disease [13], myocardial ischemia/reperfusion injury [14], and liver fibrosis [15]. Recent studies have shown that geniposide can effectively delay AS progression by reducing lipid deposition and inflammation [16–18]. However, the specific pharmacological mechanisms have not been fully elucidated.

**Fig. 1:**
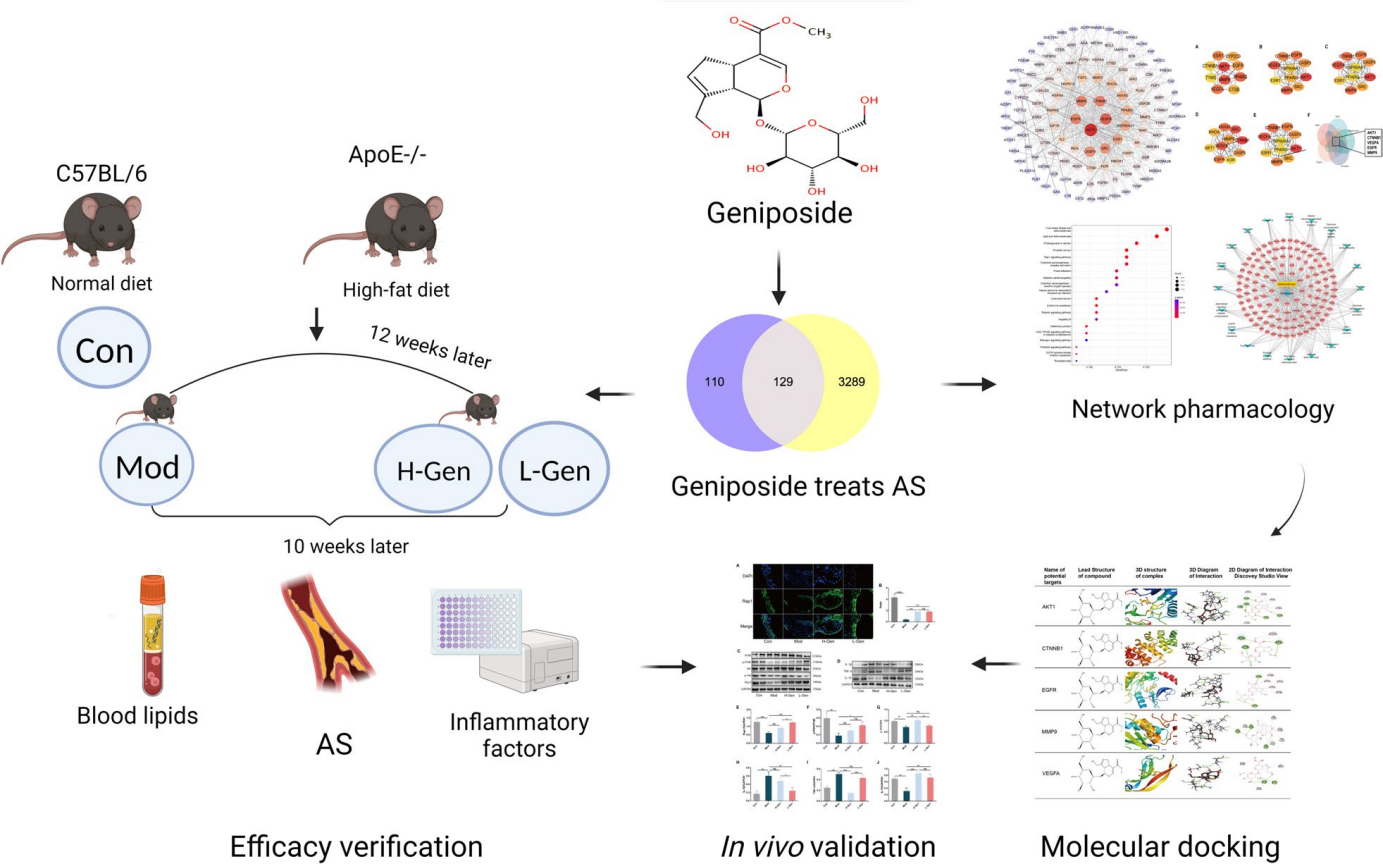
Study workflow. Network pharmacology, molecular docking, and in vivo experiments are performed to explore the pharmacological mechanisms of geniposide in treating AS

Network pharmacology elucidates the effective components and possible mechanisms of drugs for the occurrence and development of diseases from the perspective of systematic biology and biological network balance to better guide the discovery of drugs and the study of molecular mechanisms [19]. This method can effectively screen bioactive ingredients and potential targets, and has advantages in analysing the mechanism of multiple components and targets in Chinese herbal medicine [20]. Network pharmacology is increasingly used to explore the therapeutic mechanisms of TCM in various diseases. In this study, the possible mechanisms of action of geniposide were screened based on network pharmacology, molecular docking, and experimental evaluation (Fig. 1). This study provides a direction for follow-up studies of the molecular mechanism of geniposide and provides a good reference for its clinical application and pharmacological mechanism in AS prevention and treatment.

## Materials and methods

### Databases

The nine databases involved in the network pharmacological analysis are shown in Table 1.

**Table 1:** Databases involved in the network pharmacological analysis

Databases	Websites
PubChem	https://pubchem.ncbi.nlm.nih.gov/
Swisstargets	http://www.swisstargetprediction.ch/
Similarity ensemble approach (SEA)	http://sea.bkslab.org/
Pharmmapper	http://www.lilab-ecust.cn/pharmmapper/
Traditional Chinese medicine systems pharmacology database and analysis platform (TCMSP)	https://tcmspw.com/tcmsp.php
Universal protein (UniProt)	https://www.uniprot.org/
GeneCards	https://www.genecards.org/
DisGeNET	https://www.disgenet.org/
String	https://string-db.org/cgi/input.pl

### Acquisition of common targets

First, the SMILES ID of geniposide was obtained through the PubChem database, and imported into the Swisstargets and similarity ensemble approach (SEA) databases to obtain the corresponding compound targets. Second, the compound targets of the drugs were searched using the Pharmmapper, Traditional Chinese medicine systems pharmacology database and analysis platform (TCMSP), and Herb databases. The targets obtained were corrected and deduplicated using the Universal Protein (UniProt) database. Then, “atherosclerosis” was used as the key word to search the human gene in GeneCards, NCBI, and DisGeNET databases. Targets obtained from the GeneCards database were filtered according to the median score to obtain more relevant targets. The selected drug targets and disease targets were input into Venny 2.1 diagram software, and common targets were obtained, which were used as the predicted targets of geniposide on AS for pathway enrichment analysis.

### Construction of a Protein–Protein Interaction (PPI) network and selection of hub proteins

The common targets of geniposide and AS were input into the String database to construct the PPI network, and the biological species was set as “Homo sapiens” to obtain the PPI network. Cytoscape software was used for visualisation. Moreover, we applied five methods of the cytoHubba plugin, including three local rank methods (degree, maximum neighbourhood component (MNC), and maximal clique centrality (MCC)) and two global rank algorithms (betweenness and closeness centrality) according to a previous study, to identify the top five hub proteins [21]. The networks were analysed using Cytoscape3.8.0. Local methods grade hub proteins based on their proximity, and hubs are assigned a ranking based on their contact with the entire network according to global approaches. Finally, we identified the common hub proteins by combining the five cytoHubba methods.

### Construction of a component-disease-target network

To better understand the complex interactions among geniposide, AS, and the corresponding targets, we constructed a component-disease-target network based on geniposide, AS, and targets, and imported it into Cytoscape 3.8.0 for network drawing.

### Gene Ontology (GO) and Kyoto Encyclopaedia of Genes and Genomes (KEGG) pathway enrichment analysis

To describe and annotate the functions of the core targets and explore their biological processes and signalling pathways, we performed enrichment analysis on the core targets. Biological process (BP), molecular function (MF), and cell component (CC) enrichment analyses of GO were performed for the common targets of drugs and diseases and the disease targets. KEGG pathway enrichment analysis was performed on the common targets of geniposide and AS (www.kegg.jp/kegg/kegg1.html). Items with corrected *p*-values < 0.05 were screened. Cluster Profiler, enrichplot, and ggplot2 packages were installed and quoted in R 4.0.3 software to generate the bar and bubble plots.

### Construction of a component-disease-pathway-target network

The component-disease-pathway-target network file was imported into Cytoscape3.8.0 for the pathway network diagram. The multicomponent and multi-target functions of the active components of TCM in the treatment of diseases have been demonstrated more intuitively.

### Molecular docking

First, we downloaded the 3D structures in SDF format from PubChem data according to the CAS number of the small molecule. The structure was then imported into ChemBio3D Ultra 14.0. The Minimum RMS Gradient was set to 0.001, and the small molecules were saved in the mol2 format. The optimised small molecules were imported into AutodockTools 1.5.6 for hydrogenation, charge calculation, and charge distribution, the rotable key was set, and the file was saved in "pdbqt" format. The 3D structures of AKT (PDB ID: 6NPZ), CTNNB1 (PDB ID: 7UWO), EGFR (PDB ID: 8D76), MMP9 (PDB ID: 4XCT), VEGFA (PDB ID: 4KZN) were downloaded from the PDB database. Pymol2.3.0 was used to remove protein crystal water, the original ligand, etc. The protein structure was imported into AutoDocktools (v1.5.6) for hydrogenation, charge calculation, and charge assignment, atom type specified, and saved in "pdbqt" format. Docking was based on primitive ligands for those with primitive ligands. For those without primitive ligands, POCASA 1.1 was used to predict protein binding sites, and AutoDock Vina 1.1.2 was used for docking. Search space: size_x, 60; size_y, 60; size_z, 60 (each grid point is spaced at 0.375 Å); exhaustiveness, 10. Finally, the molecular docking results were analysed for interaction patterns using PyMOL 2.3.0 and Discovery Studio 2019.

### Experiment verification

#### Animals and model construction

The animal experiments performed in this study were approved by the Animal Experiment Ethical Committee of Guangzhou University of TCM (No. 20200330075). The male ApoE^−/−^ and C57BL/6 mice purchased from Guangdong Yaokang Biotechnology Co., Ltd. (Guangdong, China) were housed in a pathogen-free room at a temperature of 25 ± 1 °C in the Experimental Animal Centre of the First Affiliated Hospital of Guangzhou University of TCM. After one week of adaptive feeding, the ApoE^−/−^ mice were fed a high-fat diet (HFD, HFD formula: 21% fat, 0.15% cholesterol, 15.5% protein, and 62% common feed) for 12 weeks to construct AS models. Thirty ApoE^−/−^ mice were randomly divided into three groups (10 per group). Model group (Mod), high-dose geniposide group (100 mg/kg, H-Gen), and low-dose geniposide group (50 mg/kg, L-Gen) [18]. Ten C57BL/6 mice were fed a normal diet as the control group (Con). Next, the mice received drug intervention by gavage according to the group every day from the 13th week. The same volume of 0.9% saline was administered to mice in the model and control groups to eliminate interference from human factors. After 10 weeks of treatment, mice were anaesthetised with 1% pentobarbital by intraperitoneal injection at a dose of 50 mg/kg and then sacrificed. Blood and tissue samples were collected for pathological analysis using commercial kits and western blotting.

####  Evaluation of blood lipids in ApoE^−/−^ mice

The serum was separated and stored at − 20 °C for further examination. The levels of blood lipids, namely, total cholesterol (TC), high-density lipoprotein cholesterol (HDL-C), triglycerides (TG), and LDL-C, were detected by the Clinical Laboratory Department of the First Affiliated Hospital of Guangzhou University of TCM.

#### Histopathological examination

After being fixed in 4% paraformaldehyde for 48 h, the aortic sinus was embedded in paraffin and sliced into 4 μm sections. The histopathological morphology of the mouse thoracic aorta was evaluated using haematoxylin and eosin (HE) staining. In addition, Sirius red and Oil Red O staining were performed to evaluate the stability of atherosclerotic plaques. Immunohistochemistry (IHC) analysis was performed on paraffin-embedded thoracic aorta tissue sections to detect the protein expression of α-SMA. Images of the sections were captured using a light microscope.

#### Immunofluorescence

Immunofluorescence analysis was performed on paraffin-embedded skin tissue sections. For antigen repair, the sections were placed in 0.1 mol/L citric acid repair solution (pH = 6), heated in a microwave oven for 6 min to slightly boil, maintained in a medium heat for 10 min, and naturally cooled for 20–30 min. After washing twice with phosphate buffer saline (PBS) for 2 min, the membranes were permeabilised with 0.2% tritonX-100 for 20 min. Sections were washed with PBS and blocked in a wet box for 1 h at room temperature. After washing with PBS, anti-Ras-associated protein 1 (Rap1) (1:100, GTX101983, GeneTex) antibody was added and incubated in a wet box at 4° C overnight. The sections were rewarmed for 30 min and eluted three times with PBS. The membranes were incubated with fluorescent secondary antibodies for 2 h at room temperature and carefully protected from light. Nuclei were stained with DAPI, and fluorescence images were obtained using a fluorescence microscope.

#### Western blotting analysis

The protein concentration of the supernatant was measured using a BCA protein assay kit. Next, the proteins were boiled at 95 °C for 10 min and stored at − 20 °C after being converted into equal quality and volume. SDS-PAGE (10%) was used for electrophoresis, and the proteins were then transferred onto PVDF membranes in an ice bath at 350 mA for 60 min. Membranes were blocked with 5% skim milk at room temperature for 1 h and incubated with primary and secondary antibodies. Phosphatidylinositol 3-kinase (PI3K, 20,584–1-AP, 1:1000), protein kinase B (Akt, 60,203–2-Ig, 1:1000), phospho-Akt (66,444–1-Ig, 1:1000), Interleukin-1β (IL-1β, 16,806–1-AP, 1:1000), IL-10 (60,269–1-Ig, 1:1000), and tumour necrosis factor-α (TNF-α, 17,590–1-AP, 1:1000) antibodies were purchased from Proteintech (Wuhan, China). The antibody phospho-PI3K (AP0854, 1:1000) was obtained from Abclonal (Wuhan, Hubei, China), and anti-mouse IgG (7076, 1:3000), anti-rabbit IgG (7074, 1:3000), and β-actin (4970, 1:1000) were obtained from Cell Signalling Technology, Inc. (Beverly, MA, USA). Finally, the protein bands were visualised using Image Lab software and analysed using ImageJ software.

### Statistical analysis

All data are presented as mean ± standard deviation. SPSS21.0 software was used to analyse the data. All histogram data are expressed as mean ± standard error of the mean (SEM). One-way analysis of variance (ANOVA) and Tukey’s multiple comparison tests were used for multiple group comparisons. Comparative differences were considered statistically significant at a *p*-value of < 0.05.

## Results

### Collection of common targets of geniposide and AS

**Fig. 2:**
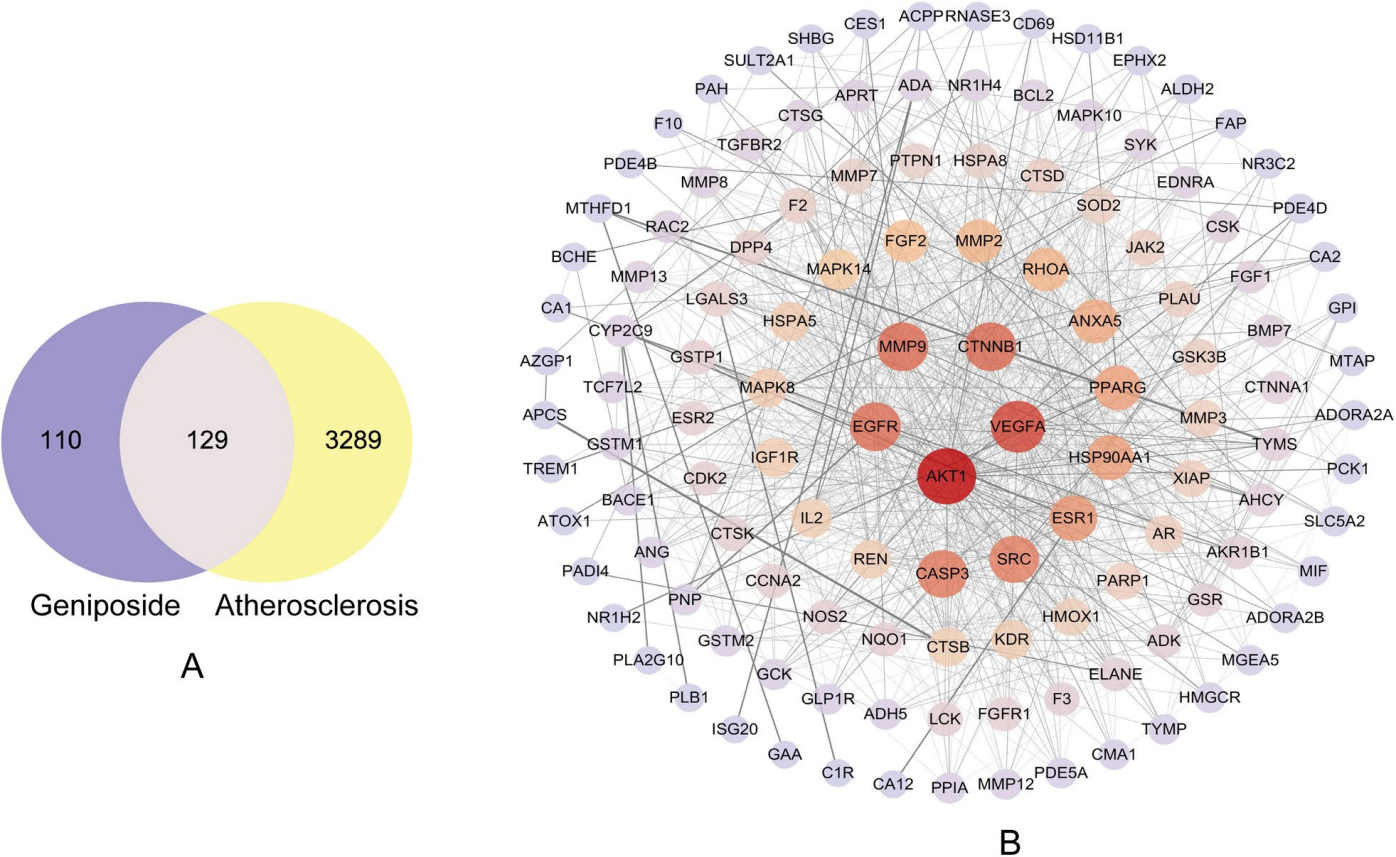
The common targets and PPI network of geniposide against AS. **A** Venn diagram of geniposide targets and AS-related genes. **B** PPI network of geniposide against AS

A total of 239 geniposide targets were obtained from public databases. Meanwhile, 3418 AS-related targets were identified. These targets were inputted into Venny 2.1 software, and common targets were obtained. As shown in Fig. 2A, 129 overlapping targets of geniposide and AS were identified; these genes are potential targets of geniposide to improve AS.

### PPI network of the targets and selection of hub proteins

**Fig. 3:**
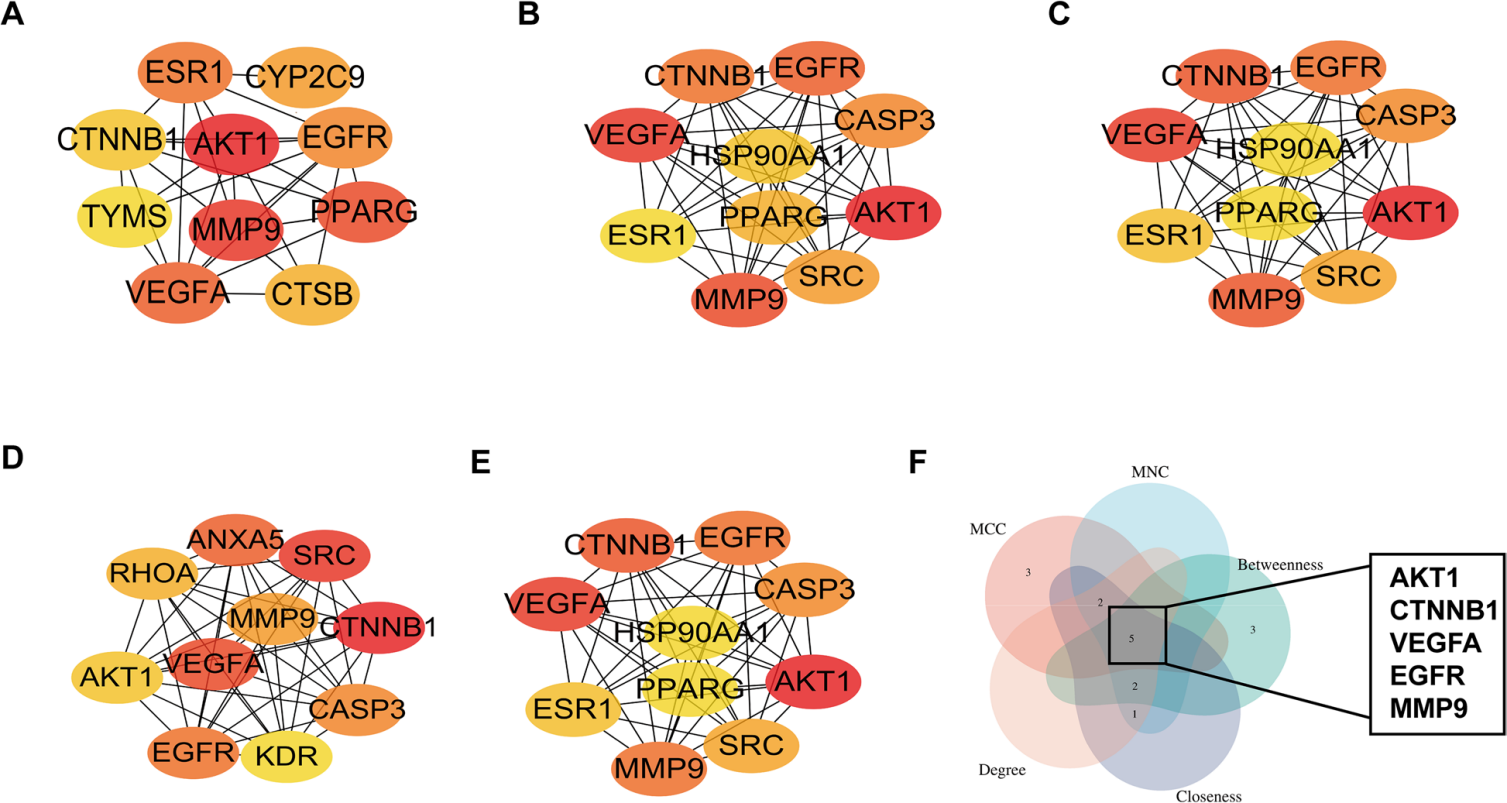
Network diagram of the core targets of geniposide against AS using five cytoHubba algorithms. **A** Betweenness, **B** Closeness, **C** Degree, **D** MCC, **E** MNC, and **F** Venn diagram of five common hub proteins

We used String database for PPI network analysis to identify the hub genes of key modules, and the results were filtered with a combined score of ≥ 0.4. The PPI network involved 129 nodes and 1048 edges, with an average degree value of 16.2. The colour and size of the nodes in Fig. 2B are adjusted according to the degree value. Hub genes are strongly associated with a large extent of association with other genes in the network, and their expression can affect the majority of the network [22]. As shown in Fig. 3, five common hub proteins, AKT1, CTNNB1, VEGFA, EGFR, and MMP9, were identified using the CytoHubba algorithm. All information on the topological characteristics of hub proteins through MCC, MNC, degree, betweenness, closeness centrality, and stress is illustrated in Table 2. These hub genes may serve as potential therapeutic targets for AS.

**Table 2:** The information of topological characteristics of the 5 hub proteins

Hub protein	Betweenness	Closeness	Degree	MCC	MNC	Stress
AKT1	1940	98	74	1.58E + 11	74	13,126
CTNNB1	485	87	55	1.95E + 11	55	4716
VEGFA	862	92	62	1.95E + 11	62	7596
EGFR	801	88	54	1.90E + 11	54	6730
MMP9	932	88	55	1.86E + 11	54	6978

### Construction of geniposide-AS-target network

**Fig. 4:**
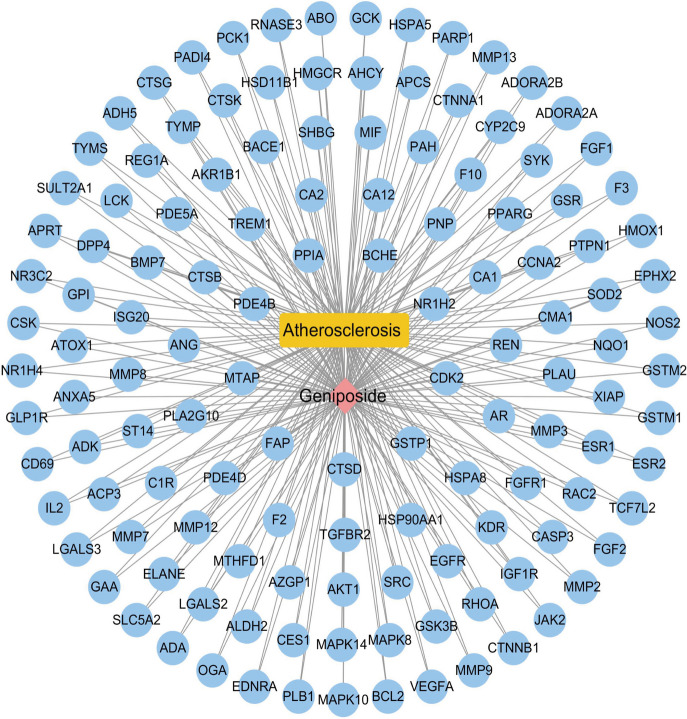
Geniposide-potential target genes-AS network. The blue node represents potential target genes; red represents geniposide, and yellow represents AS

To better understand the complex interplay between components, diseases, and corresponding targets, we constructed a geniposide-AS-target network map. All the information including geniposide, the potential targets, and AS-related genes were imported into Cytoscape 3.8.0 software to construct a network of “geniposide-AS-targets” (Fig. 4).

### GO and KEGG pathway analysis

**Fig. 5:**
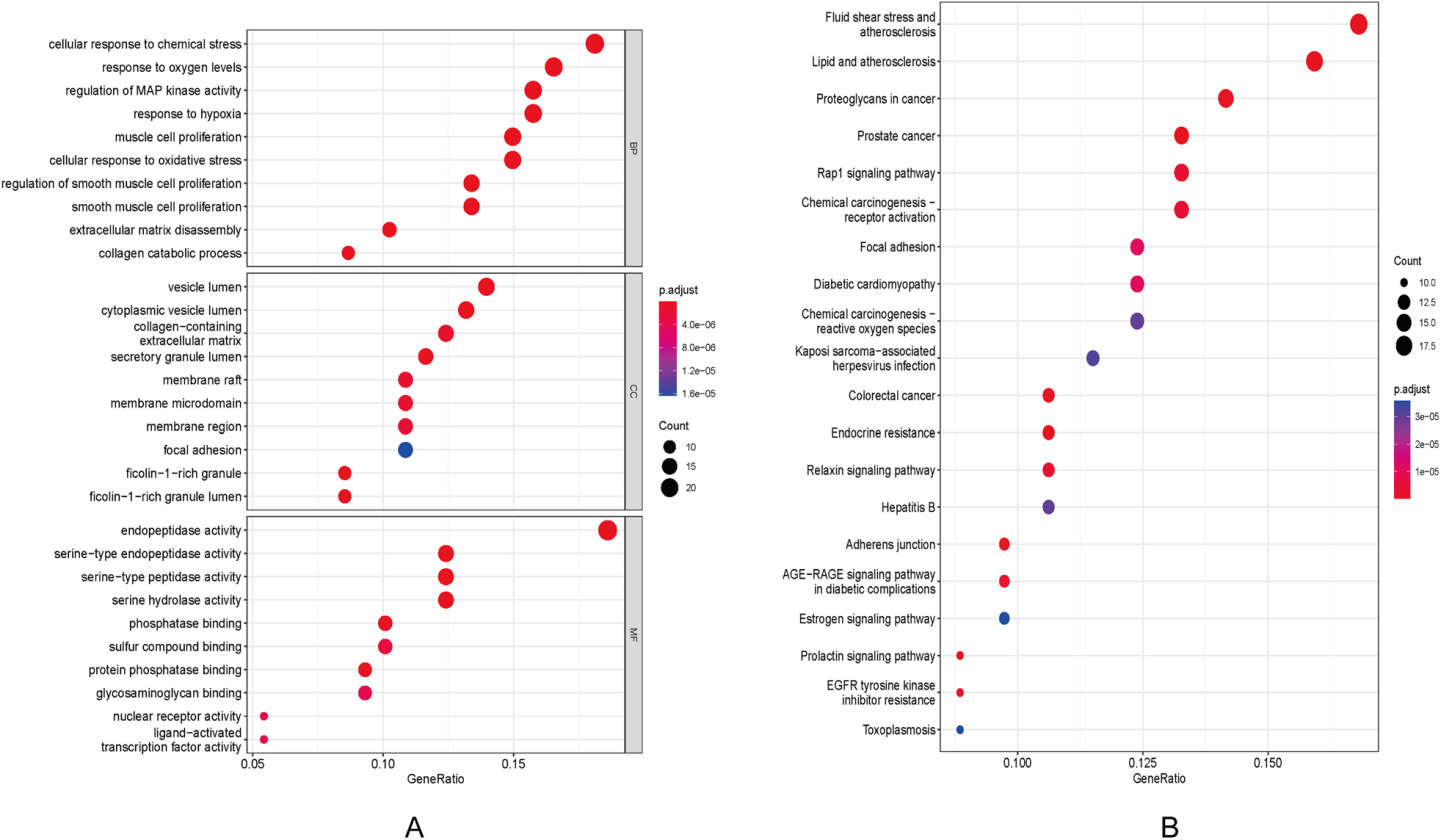
GO and KEGG terms enriched by target genes. **A** Dot plots of the top 10 enriched biological processes, cellular components, and molecular functions. **B** KEGG enrichment analysis

GO and KEGG pathway enrichment analysis were used to analyse the 129 overlapping targets involved in the PPI network to further determine the genetic function and related main signalling pathways of the target proteins. The degree of gene enrichment and significant differences in gene enrichment are represented by the gene ratio and *p*-value, respectively. The enriched terms, including BP, MF, CC, and KEGG pathways were filtered with the *p*-value, and the top 10 terms are shown in Fig. 5. According to the results, the BP terms were mainly related to the cellular response to chemical stress, oxygen levels, and hypoxia. The enriched terms in CC were vesicle lumen, cytoplasmic vesicle lumen, and collagen-containing extracellular matrix. MF enrichment terms were mainly related to endopeptidase activity, serine-type endopeptidase activity, and serine-type peptidase activity.

### Construction of geniposide–AS–pathways-targets network

**Fig. 6:**
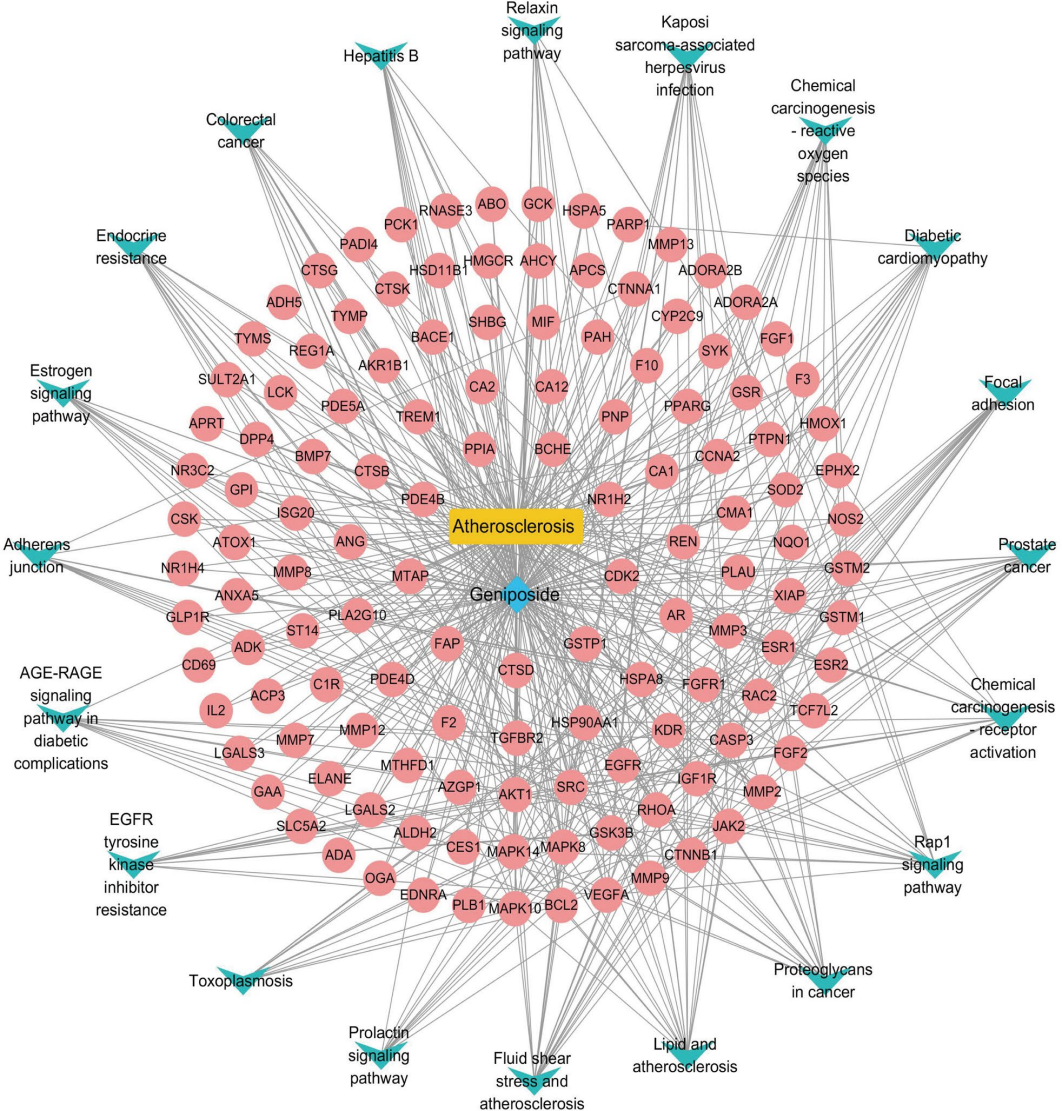
Component-disease-pathway-target network. The disease is coloured in yellow; component is coloured in blue; all the signalling pathways are coloured in green; all targets are coloured in pink

To demonstrate the multi-component and multi-target functions of the active ingredients in the treatment of diseases, the geniposide-AS-pathways-targets network file was imported into Cytoscape3.8.0 for pathway network diagram. As shown in Fig. 5B and Fig. 6, KEGG enrichment analysis revealed that geniposide affected 109 pathways in AS, including the fluid shear stress, lipid, and Rap1 signalling pathways.

### Molecular docking

**Fig. 7:**
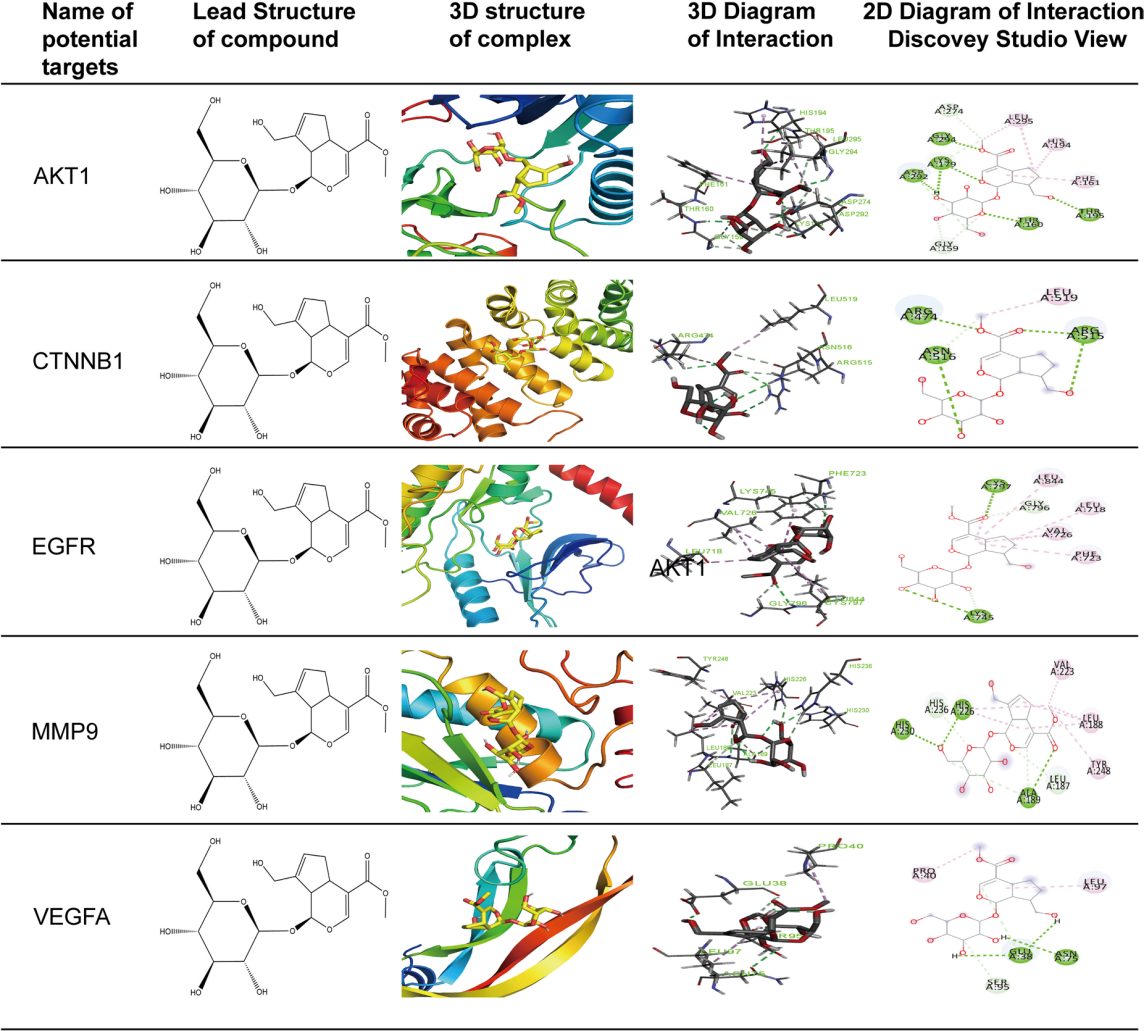
Molecular docking diagram. The results of molecular docking including 3D structure complex, 3D diagram of interaction and 2D diagram of interaction

The ability of geniposide to attach to five target proteins (AKT1, CTNNB1, VEGFA, EGFR, and MMP9) was predicted using molecular docking (Table 3). Validation of the docking tools in terms of the root-mean-square deviation (RMSD) of the co-crystallised ligand in each complex revealed that only three complexes were co-crystallised with unique ligands (CAS: 107-35-7 for 7UWO, CAS: 486-35-1 for 8D76, and CAS: 161314-17-6 for 4XCT). The docking score in this study indicates the strength of the chemical-protein binding activity: a more stable binding conformation and molecular interaction with a lower docking score [21]. Among the five examined target proteins, AKT1 (−7.3 kcal/mol), CTNNB1 (−5.8 kcal/mol), EGFR (−6.4 kcal/mol), MMP9 (−7.1 kcal/mol), and VEGFA (−5.8 kcal/mol) showed higher binding affinity than the control and co-crystalized ligands. Furthermore, geniposide bound to pocket number 1 of each protein in a suitable molecular area, as shown in Fig. 7.

**Table 3:** Docking scores of the 5 hub proteins of Geniposide against AS

Protein(PDB)	Co-crystalized ligand Docking Score	Control Docking Score (kcal/mol)	Geniposide Binding Energy (kcal/mol)	Geniposide Hydrogen bond interaction		Geniposide Hydrophobic bond interaction	
				No. of bonds	Residues involved	No. of bonds	Residues involved
AKT1 (6NPZ)	not found	-6.4	-7.3	6	GLY294, LYS179, ASP292, THR160, THR195	7	PHE161, HIS194, LEU295, ASP274, GLY159
CTNNB1 (7UWO)	-5.3	-3.7	-5.8	4	ARG515, ARG474, ASN516	1	LEU519
EGFR (8D67)	-6.2	-6.4	-6.4	2	CYS797, LYS745	5	PHE723, VAL726, LEU718, GLY796, LEU844
MMP9 (4XCT)	-6.4	-6.5	-7.1	3	ALA189, HIS226, HIS230	6	TYR248, LEU188, VAL223, HIS236
VEGFA (4KZN)	not found	-5.2	-5.8	3	ASN75, GLU38	3	LEU97, PRO40, SER95

###  Geniposide decreases body weight and serum lipid profiles in ApoE −/− mice

**Fig. 8:**
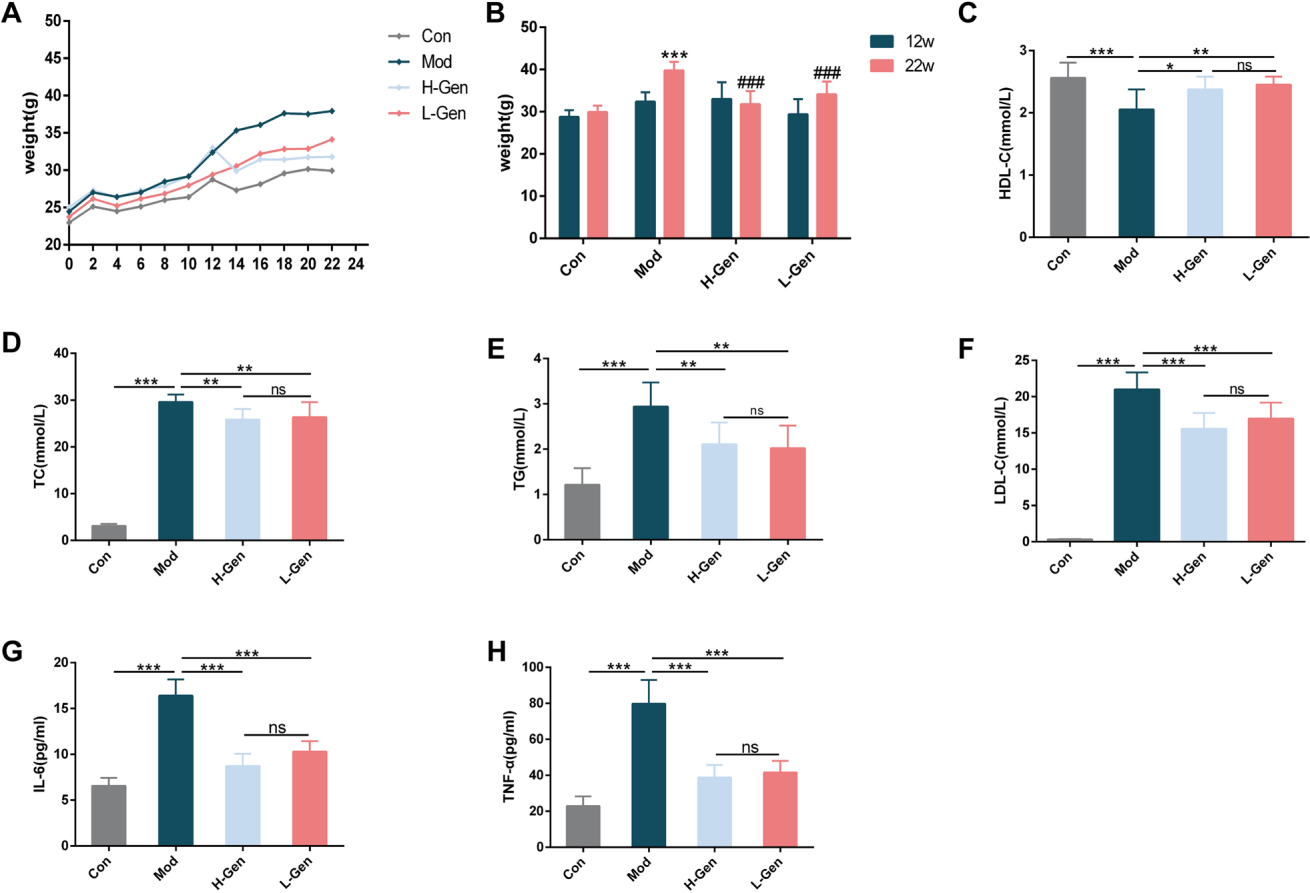
Geniposide reduces the body weight and blood lipid levels and regulates the levels of serum inflammatory factors. **A** Trends of weight gain in each group. **B** Differences in body weight between groups of mice at weeks 12 and 22; ***p < 0.001 vs control group, ###* p* < 0.001 vs model group. **C–F** Blood lipid profiles of mice in each group. **G** and **H** Expression of serum inflammatory factors in each group of mice. All column diagram data are expressed as mean ± SEM; n = 6; *p < 0.05, **p < 0.01, ***p < 0.001; ns, not significant. Con represents control group; Mod represents model group; H-Gen represents high-dose geniposide group; L-Gen represents low-dose geniposide group

To investigate the effects of geniposide on AS body weight and serum lipid profiles of HFD-fed ApoE^−/−^ mice were measured. Body weight significantly increased, and serum lipid levels were much higher than those in the control group after a 12-week HFD. Importantly, the 10-week treatment with geniposide significantly decreased body weight (Fig. 8A and B). Meanwhile, a significant reduction in the levels of TC and LDL-C in the serum and an increase in the serum HDL-C levels were observed in mice of the geniposide group. Geniposide effectively regulated the serum lipid profiles of ApoE^−/−^ mice (Fig. 8C–F).

###  Geniposide decreases the production of inflammatory cytokines in ApoE −/− mice

The serum level of IL-6 in the model group decreased remarkably compared to that in the control group. Geniposide treatment significantly decreased IL-6 levels (Fig. 8G) in the serum of mice. Compared to the model group, the geniposide groups had notably lower TNF-α levels (Fig. 8H).

###  Geniposide alleviates AS and enhances the stability of plaques in ApoE −/− mice

**Fig. 9:**
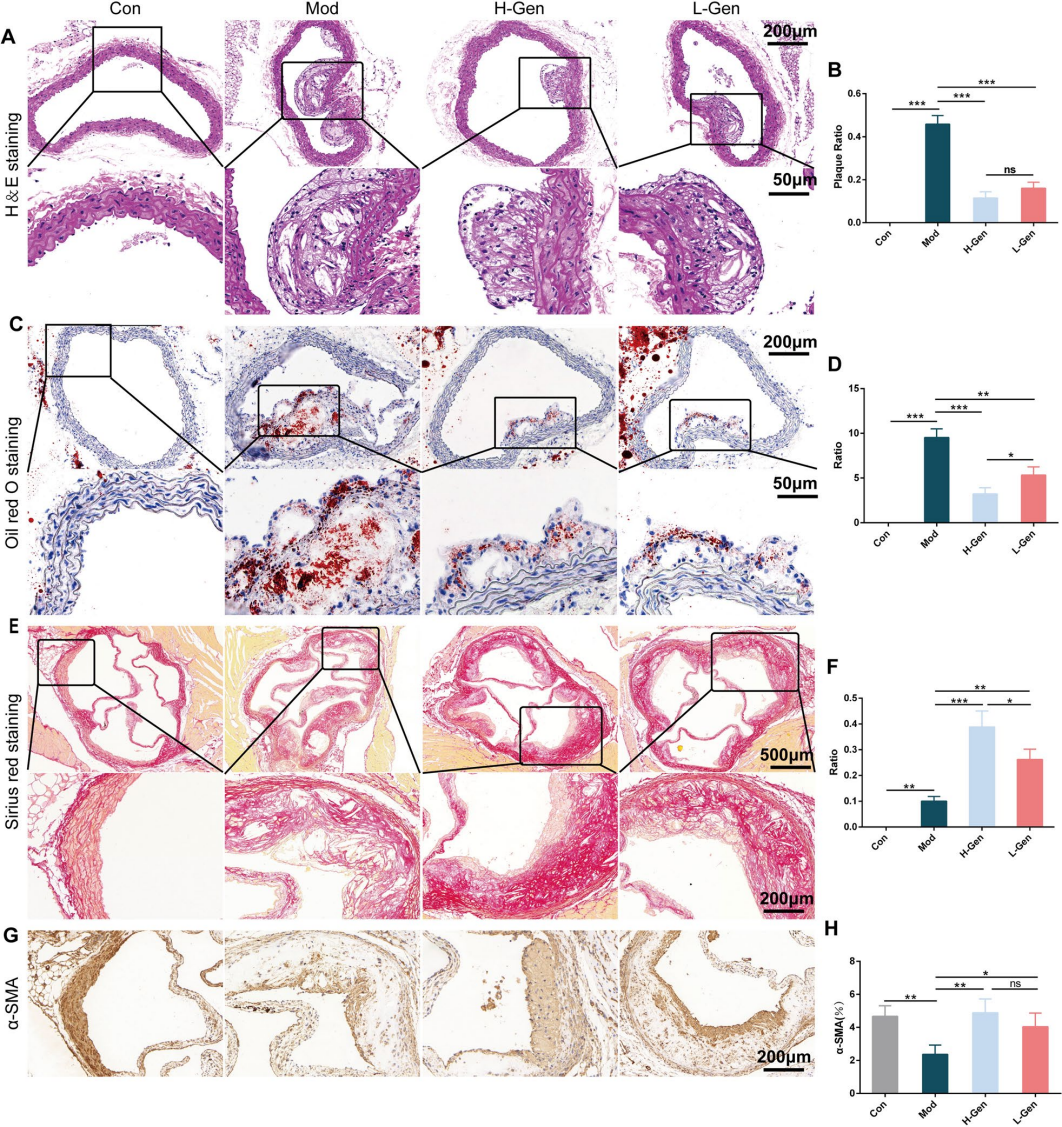
Geniposide alleviates AS and enhances plaque stability. **A–F** HE, oil red O, and Sirius red staining. **G** Immunohistochemical staining of α-SMA protein. All column diagram data are expressed as mean ± SEM; n = 3; *p < 0.05, **p < 0.01, ***p < 0.001; ns, not significant

The histopathological morphology results showed that the mice in the model group had larger plaques in the thoracic aorta (Fig. 9A and B) compared with the mice in the control group. As expected, simvastatin treatment decreased atherosclerotic lesion areas. Administration of geniposide significantly reduced atherosclerotic lesion areas and plaque size compared to those in the model group.

During AS, fibrous plaques form gradually. Plaque lipid core enlargement, inflammatory cell infiltration, fibrous cap thinning, and vascular calcification increase the risk of plaque rupture. Therefore, we examined collagen fibres, lipid cores, and inflammatory factors to evaluate plaque stability. Compared to the model group, the geniposide groups had significantly reduced lipid deposition in the plaque and a smaller lipid core (Fig. 9C and D). The collagen fibres and α-SMA protein levels increased remarkably after the intervention of geniposide (Fig. 9E–H).

###  Geniposide alleviates inflammation through the regulation of Rap1/PI3K/Akt signalling in ApoE −/− mice

**Fig. 10:**
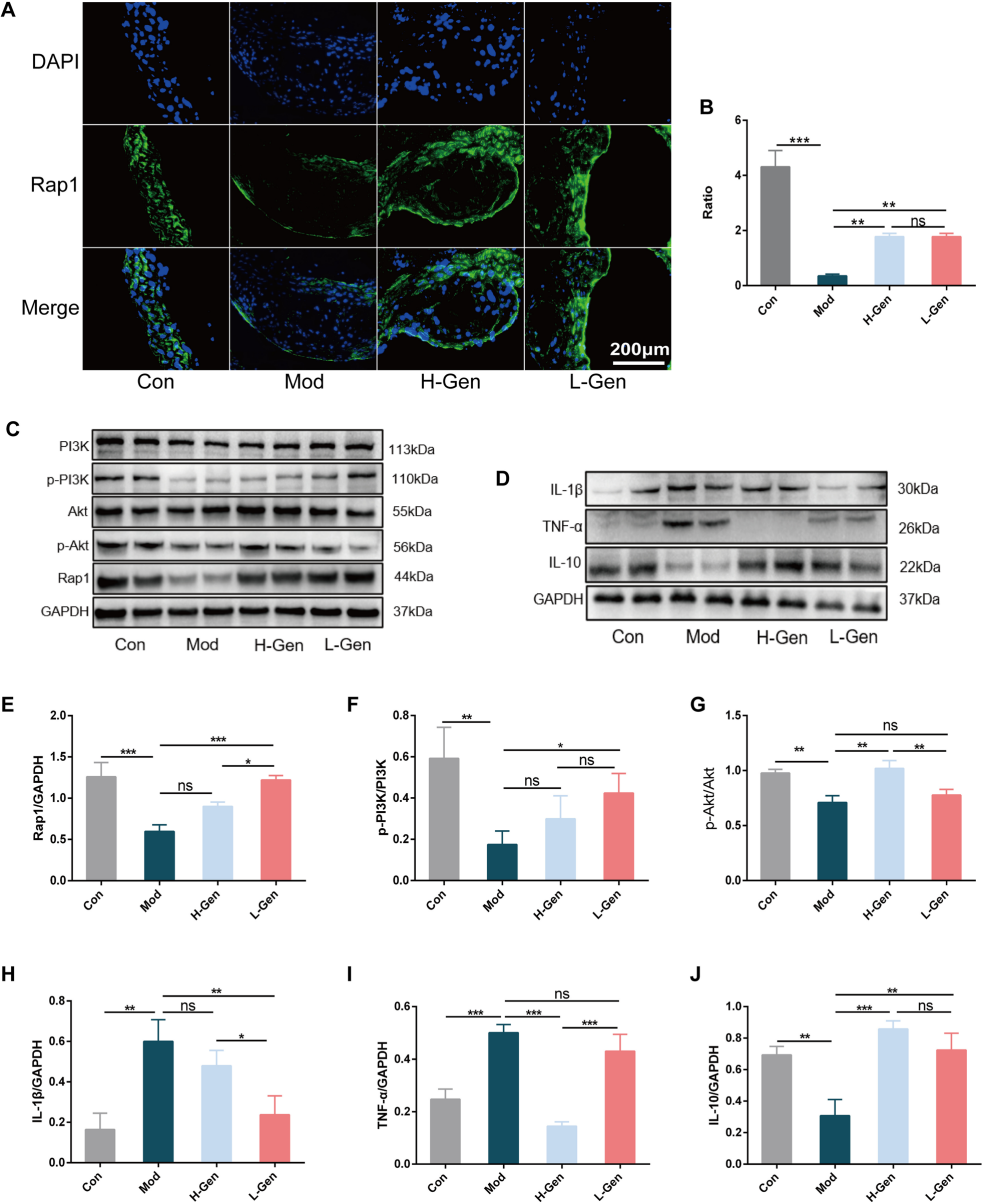
Geniposide activates the Rap1/PI3K/Akt signalling pathway and restricts inflammation in ApoE^−/−^ mice. **A** and **B** Immunofluorescence staining of Rap1 in thoracic aortic tissues of ApoE^−/−^ mice. **C–J** The relative expression level of proteins in the Rap1/PI3K/Akt pathway and IL-1β, IL-10, and TNF-α. All column diagram data are expressed as mean ± SEM; n = 3; *p < 0.05, **p < 0.01, ***p < 0.001; ns, not significant

The protein levels of Rap1 and the phosphorylation of PI3K and Akt in the thoracic aorta of the model group were downregulated compared to those in the control group. Compared with the model group, the geniposide group had remarkably upregulated protein levels of Rap1, PI3K, and Akt in the thoracic aorta (Fig. 10). Meanwhile, we measured the inflammatory condition of mice by detecting inflammatory factors in the serum and protein levels in the thoracic aorta of mice. As shown in Fig. 8G, H and Fig. 10, geniposide reduced the serum levels of IL-6 and TNF-α, decreased the protein levels of IL-1β and TNF-α, and enhanced the expression of IL-10, thus playing an anti-inflammatory role in mice.

## Discussion

AS is a serious disease with a complex pathogenesis. Modern medical equipment and drug treatments continuously optimise the treatment and management of AS; however, AS still affects the quality of life of patients and leads to death. TCM is composed of multiple ingredients with diverse pharmacological activities and various targets and pathways [23]. Accumulating evidence suggests that TCM may have excellent effects on AS treatment [24–26]. Nevertheless, elucidating the underlying mechanisms of TCM in the treatment of AS remains challenging. Network pharmacology is an organic combination of systems biology and omics that provides a direction for research on the mechanisms of complex TCM [27]. In the current study, network pharmacology combined with in vivo experiments was used to investigate the underlying mechanism of geniposide in the treatment of AS.

The targets of geniposide and AS were identified through public database search and prediction. Subsequently, 129 targets for geniposide treatment of AS were obtained by cross-mapping with AS-related targets in the GeneCards, GEO, and OMIM databases. We constructed the “geniposide-target-AS target” network topology analysis using network pharmacology. The PPI network showed that geniposide treats AS by regulating 47 core targets. The targets were sorted by degree values, and the core targets were AKT1, VEGFA, CTNNB1, MMP9, and EGFR. The gene with the highest degree was AKT1, which plays a notable role in inflammation and apoptosis in AS [28, 29]. It has been reported that VEGFA affects the development of AS by regulating lipid metabolism [30] and endothelial function [31]. Moreover, CTNNB1, MMP9, and EGFR have been shown to regulate AS by affecting the formation and stability of plaques through antioxidation and apoptosis inhibition [32, 33]. Molecular docking is an important technique for discovering new medicines and improving existing drugs. Geniposide-target docking scores were less than –5.8 kcal/mol based on the above-mentioned molecular docking results, indicating that geniposide may have strong binding activities to receptor proteins, including AKT1, VEGFA, CTNNB1, MMP9, and EGFR. Among these, AKT1 showed the best affinity for geniposide, with a docking score of –7.3 kcal/mol, which is in line with the results of a previous study [34]. GO functional enrichment analysis was used to explore the pharmacological mechanisms of geniposide. The results revealed that the key GO enrichment pathways were mainly involved in the regulation of cellular responses to chemical stress, oxygen levels, and hypoxia. These processes regulate apoptosis, tissue repair, and inflammation, thereby participating in AS pathogenesis. Pivotal targets in the PPI network were closely related to inflammation and apoptosis. Inflammation, which is the release of proinflammatory cytokines, is an important driving force of AS progression.

KEGG pathway enrichment analysis showed that geniposide acts on AS through multiple pathways, such as the fluid shear stress, lipid, and Rap1 signalling pathways. Rap is a small G protein belonging to the Ras family that has various biological functions. Recent studies have shown that Rap1 in endothelial cells improves microvascular permeability, reduces vascular hyperpermeability, alleviates tissue ischaemia after lipid peroxidation injury [35], and alleviates AS by restricting inflammation [36]. According to the KEGG pathway data, the PI3K/Akt signalling pathway is downstream of Rap1. Therefore, the Rap1/PI3K/Akt signalling pathway warrants further investigation. Further HFD-induced AS mouse model experiments indicated that geniposide is an effective treatment for AS. Geniposide reduced the formation of plaques in the thoracic aorta of mice, reduced the deposition of lipids in plaques, and increased the collagen fibre content of plaque fibre caps, thereby enhancing plaque stability. Western blot analysis revealed that geniposide could notably upregulate the protein level of Rap1, activate the phosphorylation of PI3K and Akt, promote the expression of IL-10, and inhibit the expression of IL-1β and TNF-α to protect against AS. Our findings revealed that geniposide causes a notable downregulation of Rap1, p-PI3K, and p-Akt, indicating that inhibition of the Rap1/PI3K/Akt pathway is correlated with decreased inflammation in AS. Therefore, we speculate that geniposide might exert a protective effect against AS by suppressing inflammation via the Rap1/PI3K/Akt pathway. However, this study had some limitations. Similar to most network pharmacology studies, the results predicted using this method may not be particularly accurate and require further experimental verification. We verified the predicted results, but the experiments were not sufficient; thus, additional studies are required to further delineate the mechanistic actions of geniposide on AS.

## Conclusions

This study demonstrated the therapeutic effect of geniposide on AS and preliminarily elucidated its mechanism of action. This study provides a basis for the use of geniposide in the treatment of AS and a new direction for the treatment of AS.

### Supplementary Information

** Additional file 1: Supplementary Fig. 1.** The full-length blot of GAPDH. **Supplementary Fig. 2.** The full-length blot of PI3K. **Supplementary Fig. 3.** The full-length blot of p-PI3K. **Supplementary Fig. 4.** The full-length blot of Akt. **Supplementary Fig. 5.** The full-length blot of p-Akt. **Supplementary Fig. 6.** The full-length blot of Rap1. **Supplementary Fig. 7.** The full-length blot of IL-1β. **Supplementary Fig. 8.** The full-length blot of TNF-α. **Supplementary Fig. 9.** The full-length blot of IL-10. **Supplementary Fig. 10.** The full-length blot (A) and cropped blot (B) of GAPDH.

## Data Availability

The data generated in this study are available from the corresponding author upon request.

## Abbreviations

Akt: Protein kinase B
As: Atherosclerosis
BP: Biological process
CC: Cell component
GO: Gene Ontology
HDL-C: High-density lipoprotein cholesterol
HE: Haematoxylin and eosin
HFD: High fat diet
IHC: Immunohistochemistry
KEGG: Kyoto Encyclopaedia of Genes and Genomes
LDL-C: Low-density lipoprotein cholesterol
IL-1β: Interleukin-1β
MCC: Maximal clique centrality
MF: Molecular function
MNC: Maximum neighbourhood component
PBS: Phosphate buffer saline
PI3K: Phosphatidylinositol 3-kinase
PPI: Protein-Protein Interaction
Rap1: Ras-associated protein 1
RMSD: Root-mean-square deviation
SEA: Similarity ensemble approach
TC: Total cholesterol
TCM: Traditional Chinese medicine
TCMSP: Traditional Chinese medicine systems pharmacology database and analysis platform
TG: Triglyceride
TNF-α: Tumour necrosis factor-α
UniProt: Universal Protein
