# Supplementary material for: Network pharmacology and in vivo evidence of the pharmacological mechanism of geniposide in the treatment of atherosclerosis
Source: BMC Complement Med Ther. 2024 Jan 24;24:53. doi: 10.1186/s12906-024-04356-x (PMC10807192; doi:10.1186/s12906-024-04356-x)
Supplement: Supplementary file 1 — Additional file 1: Supplementary Fig. 1. The full-length blot of GAPDH. Supplementary Fig. 2. The full-length blot of PI3K. Supplementary Fig. 3. The full-length blot of p-PI3K. Supplementary Fig. 4. The full-length blot of Akt. Supplementary Fig. 5. The full-length blot of p-Akt. Supplementary Fig. 6. The full-length blot of Rap1. Supplementary Fig. 7. The full-length blot of IL-1β. Supplementary Fig. 8. The full-length blot of TNF-α. Supplementary Fig. 9. The full-length blot of IL-10. Supplementary Fig. 10. The full-length blot (A) and cropped blot (B) of GAPDH. [file 12906_2024_4356_MOESM1_ESM.pdf]

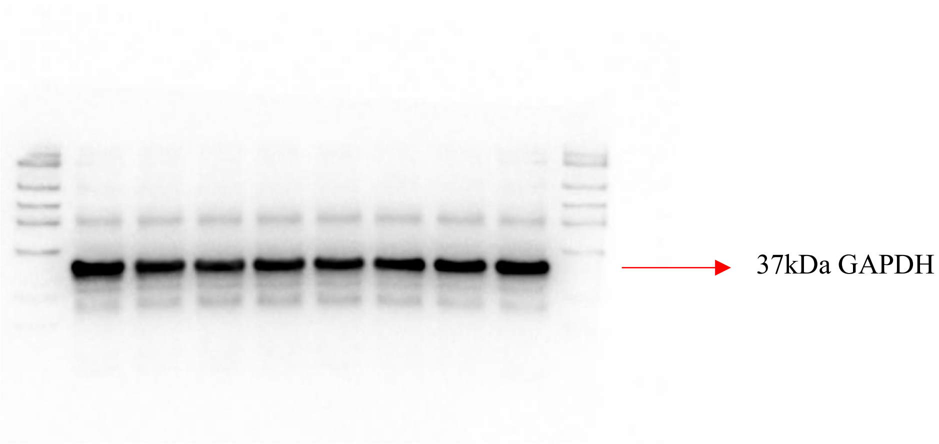

Supplementary Fig. 1 The full-length blot of GAPDH.

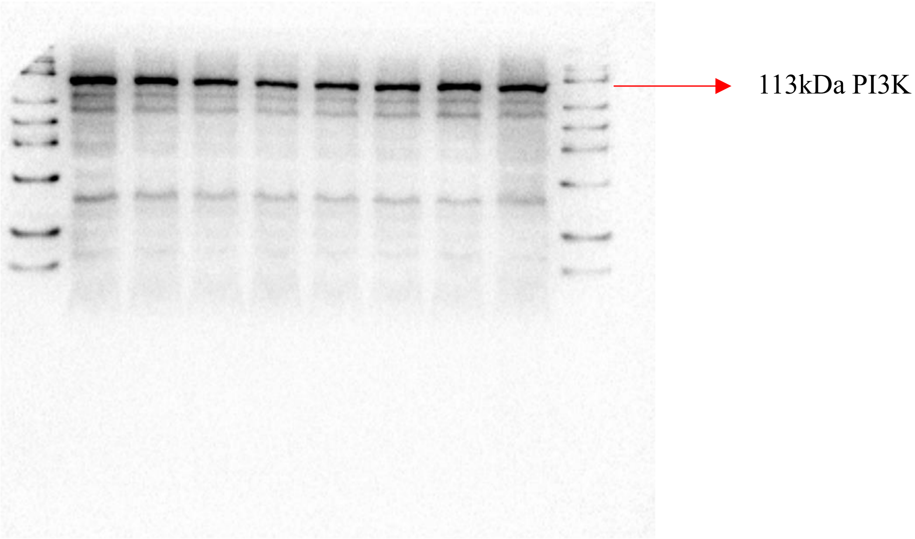

Supplementary Fig. 2 The full-length blot of PI3K.

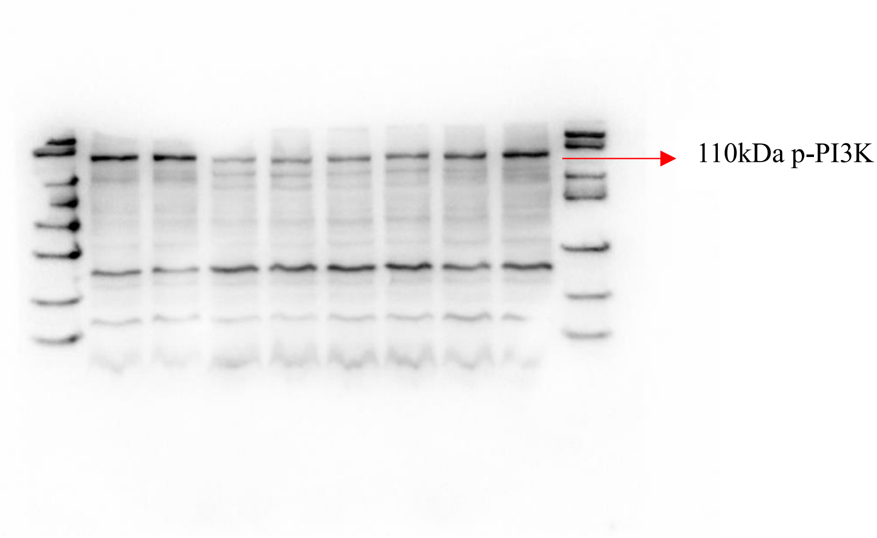

Supplementary Fig. 3 The full-length blot of p-PI3K.

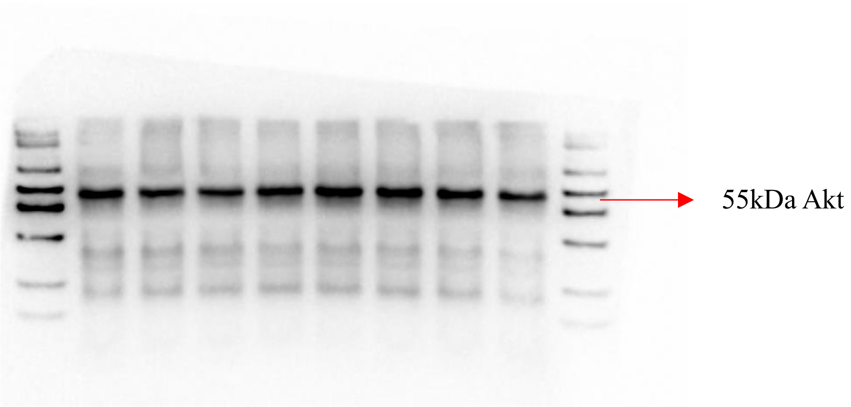

Supplementary Fig. 4 The full-length blot of Akt.

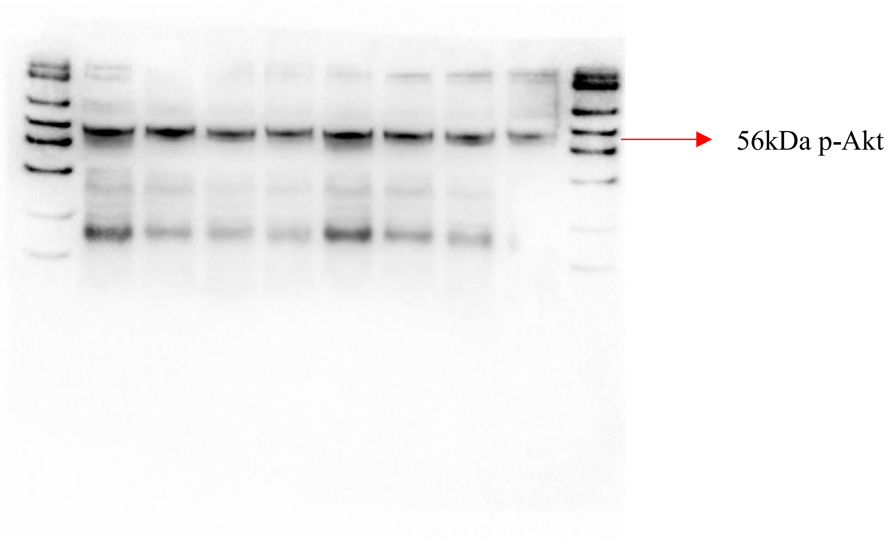

Supplementary Fig. 5 The full-length blot of p-Akt.

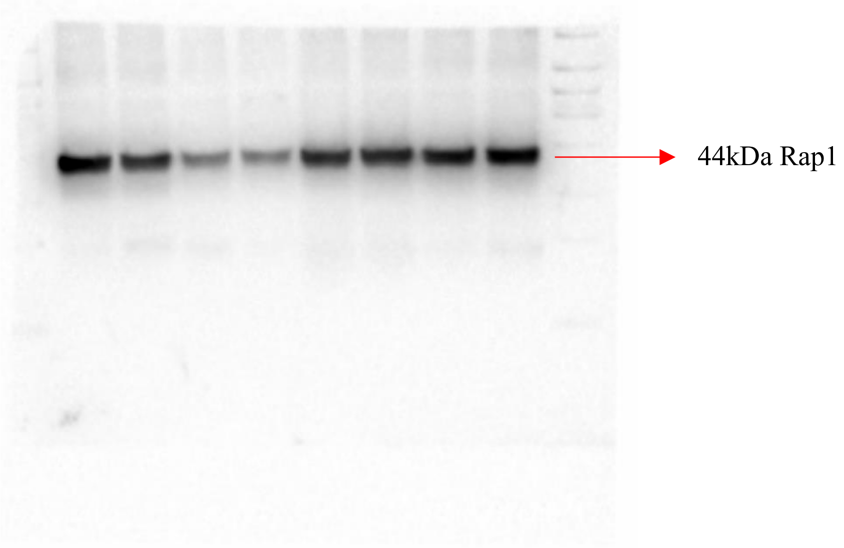

Supplementary Fig. 6 The full-length blot of Rap1.

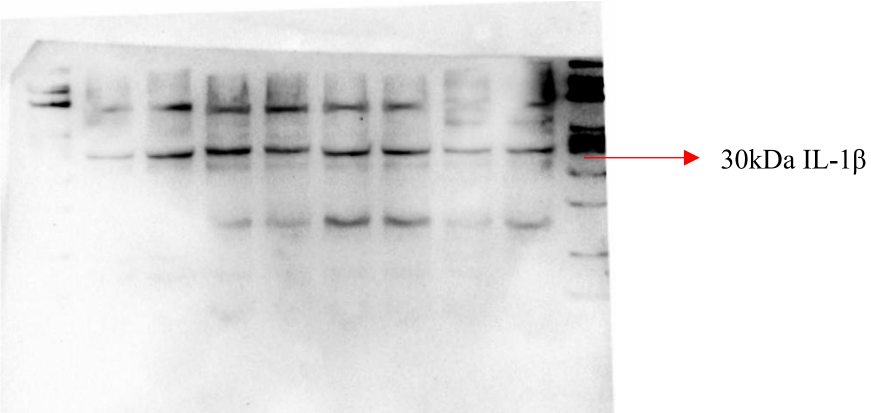

Supplementary Fig. 7 The full-length blot of IL-1β.

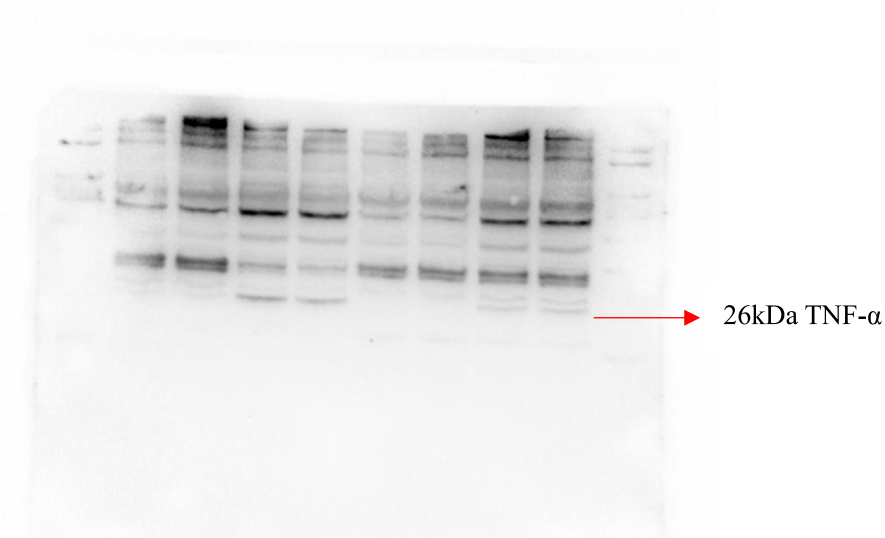

Supplementary Fig. 8 The full-length blot of TNF-α.

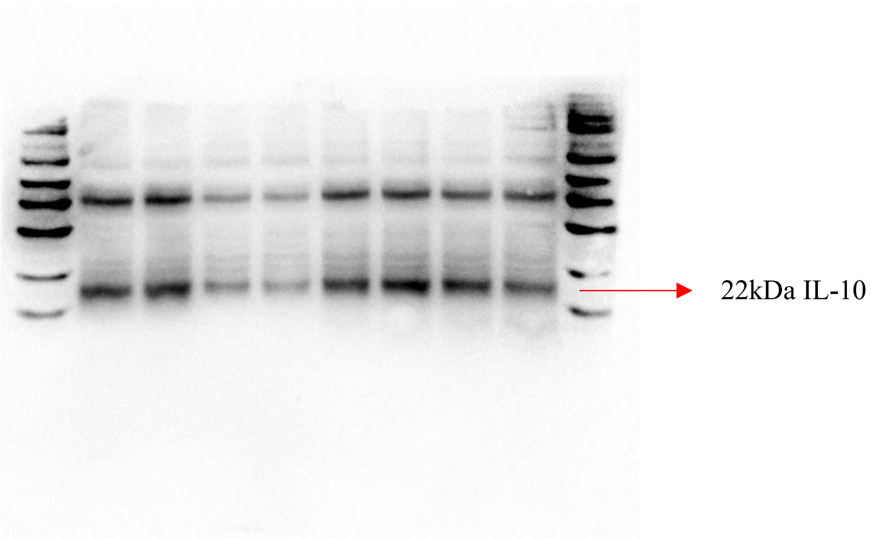

Supplementary Fig. 9 The full-length blot of IL-10.

A

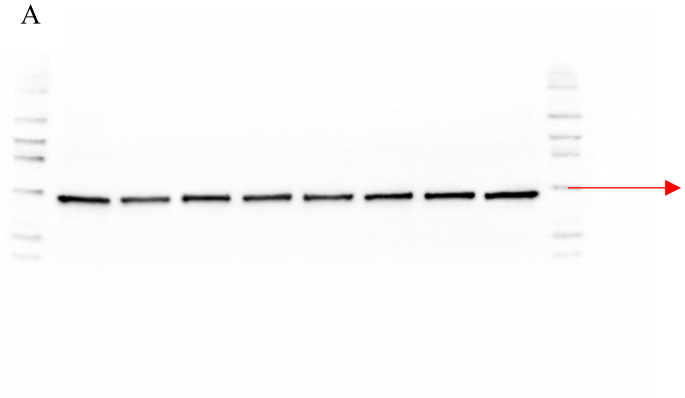

37kDa GAPDH

B

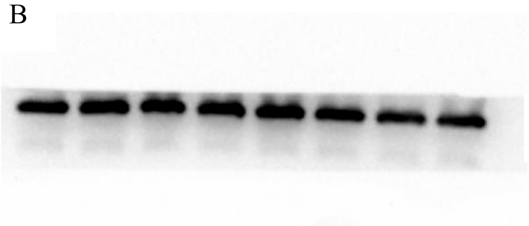

37kDa GAPDH

Supplementary Fig. 10 The full-length blot (A) and cropped blot (B) of GAPDH.
